# Conducting focus groups in realist evaluation

**DOI:** 10.1177/13563890221124637

**Published:** 2022-09-15

**Authors:** Ana Manzano

**Affiliations:** University of Leeds, UK

**Keywords:** focus groups, group interviews, middle-range theory, programme theory, realist evaluation, theory-driven evaluation

## Abstract

Focus groups are valuable tools for evaluators to help stakeholders to clarify programme theories. In 1987, R.K. Merton, often attributed with the birth of focus groups, wrote about how these were ‘being mercilessly misused’. In the 1940s, his team had conceived focus groups as tools for developing middle-range theory, but through their astonishing success focus groups have metamorphosed and are often an ‘unchallenged’ choice in many evaluation approaches, while their practice seems to provide a philosophically diverse picture. This article examines what knowledge focus group data generate, and how they support theory development. It starts with an overview of the history of focus groups, establishing a relationship between their emergence as a data collection method and the evaluation profession. Practical lessons for conducting groups in realist evaluation are suggested, while exploring how qualitative data can support programme and middle-range theory development using the example of realist evaluation.

## Introduction

Robert K. Merton, perhaps more than any sociologist before or since, was intent on reducing the gap between sociological theory and empirical research. All students of the history of sociology will be aware of the main vehicle he proposed to forge this alignment, namely middle-range theory (Merton, 1967). Much less familiar to later generations and evaluators is his work on what today we call ‘focus groups’. This article takes its inspiration from a little known and scarcely cited paper he wrote in 1987, entitled ‘The Focussed Interview and Focus Groups: Continuities and Discontinuities’. It is an urbane little essay, full of anecdote, literary illusion and self-mockery, which tells the tale of various drafts and manuscripts that had become quite unobtainable. Accordingly, he recommends as a starting point a 1946 *American Journal of Sociology* paper written with Patricia Kendall entitled ‘The Focused Interview’.

Merton’s (1987) paper is a lament on how the 1946 ideas for group interviews were becoming distorted:

In the course of time, ideas which are taken up and utilized or developed become so much a part of current knowledge, both explicit and tacit, that their sources and consequently the lines of intellectual continuity get increasingly lost to view. (p. 564)

He terms this phenomenon ‘obliteration by incorporation’. What was becoming obliterated, in Merton’s view, was the usage of the method as a tool for developing middle-range theory. He feared that its incorporation into market research, opinion polling and so on would only serve the cause of haphazard empiricism rather than cumulative inquiry.

The focus group is now one of the most frequently used methods in the evaluator toolbox (Spaulding, 2008), utilised across a variety of disciplines and in all manner of substantive inquiries (Massey, 2011). Focus groups can be one or more discussions for research purposes with pre-existing groups of people or strangers. They are presented as a set of questions at times supported by prompts such as photos, video, analogue or digital vignettes, cards, cartoons, exercises, and games (Bokhorst-Heng and Marshall, 2019; Brondani et al., 2008; Kitzinger, 2005).

Nowadays, focus groups are used in a broad range of fields and heterogeneous formats (analogue, digital, virtual, synchronous and asynchronous) (Galloway, 2011; Lobe and Morgan, 2021; Turney and Pocknee, 2005) and contexts (propaganda, public opinion research, evaluation, academic studies) (Higdon, 2020; Lunt and Livingstone, 1996; Nyumba et al., 2018), with a variety of outcomes in mind: selling (marketing) (Speight et al., 2019; Threlfall, 1999), influencing decisions (politics, health behaviour) (Dunn et al., 2020; Traugott, 2019; Wilkinson, 1998b), assessing the worth of public interventions and policies (monitoring, policy analysis and evaluation) (Kahan, 2001; Moro et al., 2007; Pact, 2014; Scott, 2011) and so on. These myriad applications, formats and platforms have diluted Merton and Kendall’s ideas for group interviews as tools to develop middle-range theory.

This article aims to examine what kind of knowledge focus group data generate, and how they support theory development. The first part of the article provides an account of the initial, halting steps to establish the focus group method and then to Merton’s attempt to formalise its role vis-à-vis theory development for evaluation purposes. It establishes a relationship between the emergence of focus groups and the evaluation profession, exploring how qualitative data have been used to construct middle-range theory in theory-driven evaluation approaches using the example of realist evaluation (RE). The second part of the article expands on how focus groups are used in the RE approach to achieve evidence-based routes for programme theory and middle-range theorisation.

Drawing on some of the original points noted by Merton in his first encounter with the emergent focus group method, the final section highlights some lessons learned when conducting focus groups in REs. These are focus groups as tools to infer causality; why conduct focus groups in REs and how many realist focus groups are enough; the ‘classroom-teachers’ cycle; and sampling participants and sub-group analysis.

## Focus groups, Merton and theory-driven evaluation

Born in the early 1940s, after World War II, focus groups seemed to remain mainly as investigation tools in broadcasting, marketing and public opinion research (Kidd and Parshall, 2000), and they did not become popular in academic and evaluation research until later in the century. In this section, an overview is provided of how the idea of using groups discussions in research and evaluation originated and how those beginnings, linked to a team of social theorists led by Robert K. Merton in the 1940s, contributed to their conceptualisation. Then the section examines how focus group success in marketing contributed to focus groups becoming a regular method in the evaluators’ toolbox. Finally, the section relates the emergence of the theory-driven evaluation approaches (Chen and Rossi, 1983, 1987) to guide evaluation efforts through a more rigorous and scientific endeavour (Donaldson, 2021) such as RE (Henry et al., 1998; Pawson and Tilley, 1997) to the revival of focus groups as originally conceptualised by Merton and others.

Originally, focus groups discussions were not part of the family of traditional social science research methods. The psychologist Bogardus (1926) had used groups to test a social distance scale, and pioneering fieldwork social scientists such as the anthropologist Malinowski (1967) during fieldwork in New Guinea and the Trobriand Islands (1914–1918); and the *Street Corner Society* author William Foote Whyte (1943) reported using group conversations for their landmark investigations. However, the visible use of focus group interviews in the social sciences starts with Merton’s team in 1941 (Liamputtong, 2011). In the 1940s, in the influential Bureau of Applied Social Research (BASR) run by Paul Lazarsfeld at Columbia University, Merton and Kendall (1946) developed what was initially called the ‘focussed group interview’ (spelled with a double s and -ed). The reader should refer to Rowland and Simonson’s (2014) pioneering account of the 34 female social scientists working at BASR. Patricia Kendall and Marjorie Fiske, co-authors of the *Focused Interview* book (Merton et al., 1956) and Herta Herzog, head of the ‘Program Analyzer Department’ were key figures in these developments.^1^

During World War II, this generic research technique emerged as a by-product of a technology designed to assess public responses to radio broadcasted propaganda. An electronic system for the quantitative recording of positive and negative audience reactions was devised by Lazarsfeld – considered by many the founder of modern empirical sociology (Jeřábek, 2001) – and called the Lazarsfeld-Stanton Program Analyzer, also known as ‘Little Annie’ (Kidd and Parshall, 2000: 295–296). Audiences in groups of 10–20 people were asked to press a different colour button when they liked or disliked what they heard on the radio. But since this mere identification was not enough to interpret the reasons behind people’s choices, Lazarsfeld invited Merton to develop a rigorous method to understand *why* people pressed different buttons and when. Merton (1987) described this inspirational moment in the following fragment:

These people are being asked to press a red button on their chairs when anything they hear on the recorded radio program evokes a negative response – irritation, anger, disbelief, boredom – and to press a green button when they have a positive response. For the rest, no buttons at all. I soon learn that their cumulative responses are being registered on a primitive polygraph consisting of the requisite number of fountain pens connected by sealing wax and string, as it were, to produce cumulative curves of likes and dislikes. That primitive instrument became known as the Lazarsfeld-Stanton program analyzer. Thereafter, we observe one of Paul’s assistants questioning the test-group – the audience – about their ‘reasons’ for their recorded likes and dislikes. I begin passing notes to Paul about what I take to be great deficiencies in the interviewer’s tactics and procedures. He was not focussing sufficiently on specifically indicated reactions, both individual and aggregated. (pp. 552–553)

Probing just for ‘reasons’, however, may have missed the more crucial patterns on why some people pressed green and some others pressed red on the same item. Probing for ‘difference’ instead, by using the comparative method as a term of reference for hypothesising the influence of group affiliations in the various participants’ behaviour, would support theory development and cumulative enquiry.

After several years of refinement, in 1946, Merton and Kendall published in the *American Journal of Sociology*, a manuscript establishing the essential features of the focussed interview as research tool that could be applied equally (albeit with some precautions) to individuals or groups. This new interview technique established a method to understand the psychological and social outcomes of mass communication as distinct from previous qualitative interviewing styles, although they ‘may appear superficially similar’ (p. 541). They highlighted four distinct and novel aspects closely related to hypotheses testing (see Box 1), where theory development for the purpose of evaluation was the prime aim of interviewing people in groups. Hypotheses were raised in order to ‘focus’ group responses and the subsequent responses lead to gradual revision and refinement of the emerging explanations.

**Box 1.** Novel characteristics of Merton and Kendall’s (1946: 541) ‘focussed interview’ technique.

1. The people interviewed have experiential knowledge of a specific social situation 2. The interviewer has previously analysed that social situation and developed hypotheses about meaning, elements and outcomes relevant to that social situation 3. The interviewer questions are prepared in advanced, taking into consideration those hypotheses to guide the data collection process 4. Although the interview is conducted by elucidating the subjective experience of participants about those social situations, this is done with two aims: ‘a) To test the validity of hypotheses derived from content analysis and social psychological theory, and b) To ascertain unanticipated responses to the situation, thus giving rise to fresh hypotheses’ (Merton and Kendall, 1946: 541).

Finally, focus groups were part of an emerging ‘what works’ agenda. When in 1941, Lazarsfeld asked Merton to accompany him to the radio studio to show him ‘Little Annie’, he had just been funded by the US government’s Office of New Facts and Figures to evaluate the effectiveness of a wartime radio broadcast. The role of the focus group was to interpret and explain the outcomes of an experimental controlled intervention. Consequently, the focus group mothers and fathers were sociologists working as evaluation consultants for radio and war opinion research.

### Focus group obliteration: Moving from marketing into evaluators’ toolbox

Data collection methods do not remain static, they evolve and self-transform and different conceptualisations overlap as practitioners use them in their investigations. Focus group evolution exemplifies a unique complex road, which leads to what Merton identified as methodological obliteration. Merton et al. (1956) had developed their focused interview techniques and ideas in the book *The Focused Interview*, which included a whole chapter about ‘the group interview’. Initially, the book did not sell many copies, but in the 1970s, as group interviewing became ‘widespread in commercial circles and is eliciting interest in the academic and non-profit sectors’ (Merton, 1987: 559–560), photocopied versions of the book were in demand. Thirty years later, a second edition of *The Focused Interview* was printed.

In 1949, the British market researcher Abrams published a detailed account of how to use the group discussion method for advertising (Catterall and Maclaran, 2007). Although US marketers had preferred individual interviews, it is reported that a fortuitous event in 1957 initiated the focus group trend. Herbert Ableson, of Opinion Research Corporation, decided to interview in a group some respondents recruited for individual interviews who were either too late or too early for their allocated interview timeslots (Goldman and McDonald, 1987). Less than a decade later, focus groups had replaced individual interviews as the preferred method of data collection in motivation marketing researchers. In fact Merton (1987), who confessed being oblivious for many years to the outstanding success of focus groups in marketing, attributed the terminological conflation of ‘focussed interviewing’ with ‘focus groups’ to an introduction written 25 years later in the book *Qualitative Research in Marketing* (Bellenger et al., 1976). He explained how in the process of diffusion of this new research method in the commercial world of marketing, much of the original conceptualisation was lost.

It was not until the late twentieth century that focus groups started to feature significantly in the qualitative social scientists and evaluators’ toolbox (Fern, 2001; Wilkinson, 1998a). Although earlier in the century most of the traditional social science qualitative research methods (individual interviews, participant observations, document reviews) were consolidated (Brinkmann et al., 2014), first editions of ground-breaking books on qualitative theory and data collection methods (Denzin and Lincoln, 1994; Silverman, 1997) did not have dedicated sections on focus groups. For example, it was only in the second edition of *Qualitative Research Theory Method and Practice* (Silverman, 2004: 177) that a chapter on focus groups was included ‘to reflect the huge gain in popularity of this method across the social sciences over the past decade or so’.

In the 1960s, large social programmes were initiated in the United States when the Congress enacted many ‘Great Society’ programmes, which generated an abundance of federal programmes and their corresponding evaluations (Alkin and King, 2016). However, a general lack of confidence in existing evaluation methods characterised evaluation writings in the 1970s and 1980s, where scholars often recommended improvements on how to conduct programme evaluations (Patton, 1982; Scriven, 1975). In the early 1980s, federal spending on social programmes rolled back (Shadish et al., 1991: 27) and with it the great hopes of improvement in effectiveness (Weiss, 1997), with many evaluations being smaller and internal (Shadish et al., 1991: 27). Focus groups replaced and/or complemented interviews and follow-up mail or telephone surveys. They were seen as having several advantages over other methods for evaluating client perceptions and opinions, such as cost-effectiveness and flexibility, while capturing the many complexities in social programming (Magill, 1993). Although for around 50 years focus groups had been used by industry to evaluate public reaction to services and products (Magill, 1993: 107), their increased use in social science research within the political context of the 1980s legitimated them in social welfare research and consequently in programme evaluation.

### Using qualitative data to construct middle-range theory in realist evaluation

At end of the 20th century, new developments in the evaluation profession inadvertently revived the original ambitions of Merton et al. for focussed theory-driven interviewing. The 1990s saw Chen (1990), Weiss (1997) and other evaluation scholars grounding their work in scientific knowledge by using conceptual frameworks and testing and developing programme theory as the key aim of their evaluation approach. In 1991, Shadish et al. had argued for evaluation theory to be closer to empirical research, describing theory-driven evaluation as ‘a comprehensive attempt to resolve dilemmas and incorporate the lessons from the applications of past theories to evaluation practice’ (Donaldson, 2021: 6).

In Europe and the United States (Henry, 2016), the realist approach to evaluation science emerged as a form of theory-driven evaluation. The European brand was consolidated by the work of Pawson and Tilley (1997) and their context–mechanism–outcome configurations (Pawson, 2006b). In the United States, Henry et al. (1998), sharing the same philosophical roots, worked with a less prescriptive approach to illuminate underlying mechanisms. The writings of Pawson (2000) on evaluation theory followed on explicitly from Merton’s (1967) middle-range theory work while operating within a scientific realism positioning that recommends ‘belief in both observable and unobservable aspects of the world described by the science’ while ‘epistemologically, realism is committed to the idea that theoretical claims [interpreted literally as describing a mind-independent reality] constitute knowledge of the world’ (Chakravartty, 2013). The realist premise is that the real (mechanisms), the causal (events which may or may not be observable) and the empirical (evidence of experiences and observable events) are elicited through a series of hypotheses, which are tested, refined and tested again through an ongoing iterative process.

Following this philosophy of science, Pawson (1996) in *Theorising the Interview*, positioned theory development at the forefront of the conduct of the semi-structured qualitative interview for evaluation purposes. The evaluator’s theories and not the subject’s perspectives are the subject matter of the interview because social betterment policies, programmes and interventions are in fact ‘theories’ (Vaessen and Leeuw, 2011). With this premise and purpose, Pawson proposed conducting qualitative interviews, by placing evaluators’ theories before the interviewee for them to comment on and providing theory refinement. This process, called the learner-teacher cycle, starts by the interviewer teaching the respondent ‘the particular programme theory under test’, who then is assumed to be able to teach the evaluator back about hypotheses components ‘in a particularly informed way’ (Pawson and Tilley, 2004: 12). The ‘cycle’ here refers to the interchangeable roles between the interviewer and the interviewee during the communication process of dyadic thinking. In 2016 Manzano, building up from Pawson’s paper, proposed three distinct and interlinked phases in RE interviews: theory gleaning, theory refining and theory consolidation. Despite using qualitative enquiry, REs distinguish themselves from constructivist investigations because design and fieldwork activities theorise, test those theories, refine and test again in an iterative process for the purpose of cumulative enquiry.

Up to the time of writing, RE scholars have mostly reflected on the role of individual interview techniques (Brönnimann, 2022; Mukumbang and van Wyk, 2020; O’Rourke et al., 2022) in theorisation, with focus groups not being mentioned or discussed methodologically as a distinct social research method (Smeets et al., 2022). In the following section, four key lessons are identified to understand how the focus group dynamics influence the process of theory development in the realist approach to evaluation. Those conducting focus groups may want to use these lessons as starting points for further development.

## Developing theory with the help of group discussions in realist evaluation

Evaluators aiming to embark in realist studies need to be aware of what their ontological positioning means and a clear epistemological rationale as to why (Parker and Tritter, 2006) they are collecting focus group data in particular. This section provides some practical tips for conducting group deliberations in RE studies. These were identified and developed through my own evaluation practitioner knowledge. This knowledge was supplemented with a scoping review of peer-reviewed published studies (1997–2019) described as REs) or realist syntheses (RSs) in the titles and abstracts and using focus groups (see Supplementary Table S1). Forty studies were purposively selected to reflect a range of study designs, comprising REs (*n* = 20) and RS (*n* = 20) and including five study protocols for each of those groups (RS and RE). The full text of the papers was examined to understand how and when group discussions are used in theory development in RE.

## Choosing realist group discussions as evaluation tools to infer causality

When in 2001 Fern proposed using focus groups to validate ‘theoretical notions’, this was seen as almost revolutionary. Hurworth’s (2003) review of Fern’s book highlighted how radical this was at the time:

In amongst such text there are elements of surprise where some quite radical ideas are presented. For instance, he advocates some departures from traditional ways of dealing with focus groups such as organising groups without moderators, focus groups with informal moderators or holding groups solely for validating theoretical notions. (p. 39)

Despite Merton et al.’s conceptualisation, at the beginning of the 21st century using focus groups outside the interpretivist or positivist paradigms was still perceived as a clear deviation from methodological norms.

RE differs from other types of theory-driven evaluation approaches: the explicit focus is on identifying causal processes by examining how programme outcomes are generated by underlying mechanisms, which are enabled/disabled by different contextual circumstances. The realist approach to evaluation is rooted in a specific philosophy of science, ‘scientific realism’ (Pawson, 2006b), and this philosophy should penetrate into formal or informal data collection tools employed in these investigations. Therefore, evaluation design decisions should reflect this aim and, consequently, realist evaluators should consider planning focus groups to help substantiate causal claims.

Realist evaluators run focus groups because they are after the key theory-driven feature that makes them unique: ‘group intelligence’ (or ‘group reasoning’). They examine theory-relevant responses for sub-groups of populations impacted by the programme, while understanding that the context of thinking in a group is different from the context of individual reasoning. They see that difference as conducive to identifying that elusive causality often hidden in underlying mechanisms (Westhorp, 2018). Consequently, focus group data are analysed with causality (and not experience or thematic description) at the centre of the analysis. In realist studies, theme and theoretical saturation are often not sufficient analytical tools to infer causality, although they can be useful in the early stages of theory gleaning.

Instead, retroduction, that is, ‘going back from, below, or behind observed patterns or regularities to discover what produces them’ (Lewis-Beck et al., 2004), is the main realist analytical strategy. This should be pursued through many avenues, with group reasoning being one of those. Since many scholars such as Merton (1987) himself do not think focus groups have enough standalone causal power to be used as evidence, it is important that they are integrated in mixed-methods designs to support triangulations and interpretations. If they are run on their own in smaller realist projects, they can be treated as one of many nuggets of evidence (Pawson, 2006a) to be sustained, refined or discarded with the support of social science theory.

In addition, there are other specific features of the research setting in which focus groups occur that make them potentially valuable for realist inquiries. For example, observing group relational interactions (e.g. non-verbal communication, seating arrangements, participation and leadership behaviours) could help gleaning ideas around mechanisms. Brown (2015) explained how conducting focus groups in naturally occurring settings can potentially enable complex social data to surface. Relevant features of contexts could also be elicited and/or refined when conducting focus groups in specific locations and field settings. For both interactive and substantive content, RE studies should report how the theoretical knowledge flows through different methods of data collection and within and across group encounters, identifying how group discussions are located in the distinct realist methodological phases (knowledge elucidation/gleaning, refinement, consolidation).

## Why conduct focus groups and how many realist focus groups are enough?

In small-scale evaluations, the reasons to choose whether to interview individuals or groups are often purely logistic. However, some issues are better discussed individually through in-depth interviews for ethical, privacy and/or theoretical reasons (e.g. to consolidate specific hypotheses/ programme theories). Individual interviews and focus groups are both useful to explore propositions that will be tested and refined with other data. They are similar methods and much of the advice for conducting individual interviews also applies to focus groups. Nevertheless, they are distinct in many other ways. For example, a homogeneous group of people in a focus group may find it easier to talk to one another and bounce back ideas about programmes, interventions and topics they all have similar expertise in. Heterogeneous groups of participants can compare responses with each other and expose, for instance, the lack of consensus in complex transdisciplinary programmes characterised by multiple stakeholders with competing interests. While individual interview data have been known to encourage the ‘risk of “armchair” theorizing about the causes of such difference’ (Kitzinger, 1994: 117), in groups, differences can be examined ‘in situ’ and this allows researchers to explore and observe how people theorise their views ‘in relation to other perspectives and how they put their own ideas ‘to work’’.

There is no agreement in the qualitative research methods literature on the optimum number of focus groups (Guest et al., 2017). Methodological studies aiming to establish ideal sample sizes often employ average calculations of aggregated published studies that use ‘theoretical saturation’ as their primary analytical strategy. However, as Guest et al. (2017) pointed out, by definition theoretical saturation is ineffective for estimating sample sizes prior to study implementation, since it can only ever be determined during or after data analysis. Significantly, realist evaluators do not refine or discard their hypotheses through conceptual theoretical saturation, but through relevance and rigour while digging for nuggets of evidence in other mixed-methods sources of data (Pawson, 2006a).

In essence, for realist evaluators, samples can only be weakly elaborated before fieldwork commences (Emmel, 2013), with rough ideas being clarified during fieldwork. RE sampling strategies should aim to test hypotheses about programme complexity. These may be about evaluation sites, population groups, implementation barriers, facilitators and so on. As evaluators become knowledgeable of programme successes and barriers, theories will start to develop shape and the approximate number of feasible groups discussions can be established and then pursued. Nevertheless, expert evaluators know very well that focus groups are notoriously onerous to organise and even when recruited, some group members are also notably difficult to gather in the same room. Consequently, theoretical hurdles, iterations, contingencies and last-minute practical decisions can impact how many focus groups can be conducted. These leave evaluators with little control over final number of focus groups and of attendees per group. In summary, as with the realist interviews, the importance is not on ‘how many’ groups of people we talk to but on ‘who’, ‘why’ and ‘how’ as it will be further explained later on in this article.

In the RAMESES quality standards for RE (Wong et al. 2017), the ‘Data collection methods’ standard states that methods must be explicitly consistent with realist methodology (e.g. realist interviewing) but does not distinguish between interviews and focus groups. In practice, RE studies tend to use more interviews than focus groups, often combining them, using the same topic guides for both, and not clarifying whether their data emerged from individual or group conversations and how these impacted different causal explanations. These are, however, distinct data collection methods as will be explained in the following section.

## The classroom-teachers cycle: Talking to groups like a realist

In 1996, Pawson proposed a key relational distinction when conducting qualitative interviews, consisting of placing evaluators’ theories before the interviewee for them to comment on with a view to providing theory refinement. The learner-teacher cycle starts by the evaluator teaching the respondent ‘the particular programme theory under test’ who then ‘is able to teach the evaluator about those components of a programme’ (Pawson and Tilley, 2004: 12). The ‘cycle’ here refers to the interchangeable roles between the interviewer and the interviewee during the communication process of dyadic thinking. This innovative technical advice to realist interviewers, however, has not been developed for the specific context of group settings, where thinking and dialogue are no longer restricted to two people but to an unpredictable set of people gathered together. In the following section, this dialogical plurality is discussed while reflecting on how this process is distinct in group deliberation encounters aiming to construct theoretical notions related to causality.

### The ‘deliberator’ and the classroom-teachers cycle

Realist evaluators do not hide their knowledge from the groups they are consulting; they ‘deliberate’, sharing their knowledge as a strategy to get group reasoning going, so they can together uncover the elusive hidden causal processes typical of complex programmes. For this reason, realists do not ‘facilitate’ or ‘moderate’ groups discussions. Instead, they cautiously share their tentative hypotheses, hoping that the nuggets of rough evidence will be challenged, refined or discarded by participant’s own knowledge of the programme.

Teachers and educators know how hard it is to create a culture of learning in group settings. When the conversational setting is changed from an individual to a group discussion, then the group is there to deliberate on the evaluator’s theory. This deliberation consists of the classroom becoming the teacher by helping the evaluator to confirm, falsify or refine those hypotheses. It can be helpful if before meeting the group, the realist evaluator prepares a list of potential tentative causal hypotheses or their components (Cs, Ms, Os, CMs, COs, MOs, CMOs) that could be shared loosely during group deliberations, asking for examples where this may or may not apply. These hypotheses are often initially gleaned in previous data collection methods, by experienced evaluators using their wealth of knowledge from evaluating similar programmes, or by digging in the general social science literature.

While many textbooks talk about facilitators ‘controlling the group’, for realist deliberators this means being in control of the literature and the theories that they need to refine with the help of group intelligence to provide ‘assisted sensemaking’ (Mark et al., 1999). While ‘realist interviewing assumes that people know different things according to their roles’ (Manzano, 2016), a realist focus group assumes that when those different people are in the same room, they will say different things and they will not necessarily agree. It is not consensus that is pursued but disputation, contradictions and disagreements. This advice repositions the role of the evaluator and also the role of the group, that is no longer perceived as a beast with wild ideas and behaviours to be controlled to achieve consensus and representativeness. Instead, the realist focus group aims to be a unique classroom of students and teachers who are expected to disagree and challenge each other. This provides evaluators with examples, exceptions and contradictions that will provide a rich sub-set of possible causal explanations or circumstances to be tested.

### Sharing programme theories with groups of stakeholders

In most of the purpose-built focus group facilities around the globe to run marketing and polling research focus groups, there is a mirrored window separating participants and moderators from the back room. This ‘client’ room is often described with observation capacity seats for 10–20 people, facilitating clients’ and researchers’ invisible observations of the group discussions. An intriguing phenomenon occurs when earpieces are used to communicate and give instructions to moderators, ‘often, the participants begin to talk to the mirror rather than to the moderator, since they feel the more important people are behind the mirror’ (Greenbaum, 1998: 50).

In realist studies, it is the more important evaluator’s theory metaphorically hidden behind the mirror that focuses the group conversation. Contrary to the classic advice, in REs, the theory is brought to the front of the group. For example, in an RE of a maternal health programme in Nigeria (Mirzoev et al., 2015), women who had used the primary health facilities were consulted to explore how conditional cash transfers (CCT) influenced decision-making on health utilisation for pre-natal, delivery and post-natal care. When the realist evaluator presented to the group (‘the classroom’) the tentative theory that CCT made women more confident to attend the healthcare facilities, this was immediately challenged by participants who became ‘teachers’ to the evaluator. First, by thinking about their own diverse reasoning to attend the health centre, and second, by looking for nuggets of evidence in the stories of the other people they knew that could help sustain or discard their own reasoning.

CCT programme theories assumed that money was a key mechanism for pregnant women to access healthcare, but this programme theory was discarded by the group deliberation who instead referred to the safety of the newborn as being the key driver for women’s choices. The presence of a physician (implying a safer delivery) in the primary healthcare facilities is a rarity in Nigeria (Villar Uribe et al., 2018) and this seemed a key driver for some women (i.e. those with previous health conditions, those who can afford private healthcare). This new group intelligence helped explain differences in healthcare utilisation at pre-natal, delivery and post-natal visits, since in some of those (e.g. postnatal child immunisation visits), the need of a physician to ensure safety may vary.

This example demonstrates how group intelligence can often slowly discard the simplistic explanations carried in many programme theories and by many evaluators at the early stage of data collection. This is done through a process of contrasting, comparing and sharing notes with the group who may or may not build consensus. Kitzinger (1994, cited in Parker and Tritter, 2006: 26) refers to this unique group dynamic as a ‘synergy’ between participants where group intelligence grows momentum to explore meanings alongside reporting their own individual experiences. In the case of realist group deliberation, the evaluator is included in that synergy, also challenging explanations, comparing and sharing notes (as tentative programme theories or Cs Ms Os) with the group.

## Sampling participants and sub-group analysis in realist focus groups

In many quantitative studies, sub-group analysis is tackled with extreme caution. In fact, this is often avoided or dismissed because drilling down into programme outcomes in many specific sub-groups can lead to such small sample sizes that claims will be made on the basis of non-representative statistical results. One could say that the fear of false positive errors drives the analysis and often only outcomes for large sub-groups are examined or reported. On the contrary, sub-group analysis is always an aim of realist investigations because they assume that programmes have different outcomes for different groups in different circumstances. Realist evaluators think about sub-groups who respond or not, who have this or that kind of barrier, impacting these or those programme outcomes, generating many sub-sets of known and unknown contextual circumstances. Drilling down the rabbit hole of sub-groups releases explanations needed to support theory development. However, programmes impact an infinite number of sub-groups and questioning all those groups is often an impossible task because many multi-sets of infinite predictable and unpredictable theory-relevant characteristics may apply. In well-resourced evaluations, questions about how programme theories are affected by different circumstances ‘should be asked repeatedly for different groups (e.g. children, parents, workers, the community as a whole) until the range of outcomes has been identified’ (Westhorp and Manzano, 2017: 1). This is, of course, unfeasible for many low budget, rapid, low resourced evaluations.

Realist focus groups can be an excellent tool to collect nuggets of evidence on how programmes impact sub-groups disparately and/or how mechanisms may or may not be triggered in certain circumstances. These could be more or less likely to be triggered for certain sub-groups with certain sub-characteristics. Sub-group voices are not collected because they ‘represent’ all programme participants but because they provide comparative data to inspect contexts (data from different locations, time periods, similar programmes, etc.). They may represent the voice of many contextual circumstances of many sub-sets of possibilities, which are likely to impact the implementation (and outcomes) of complex social programmes. It is not sub-group voices but the differences between sub-groups that drive the investigations. Consequently, focus group participants are not recruited on the basis of demographical characteristics but on how they may support in developing theory. Theory-relevant (as opposed to ‘variable-relevant’) groups of stakeholders help explore differences in how people respond to a programme.

‘Reference group theory’ (RGT) – another key contribution of Merton (1967) to social theory and one of his most developed middle-range theories – becomes a useful tool to explore the benefits of conversations with groups for theory-development by supporting sub-group examination through comparative analysis. RGT (see Table 1) supports causal explanations by focusing on the role of group affiliation. The focus on ‘for whom’ these hypotheses work and why is a useful instrument for theory development; those who aspire, those who are indifferent or those who are motivated not to belong, have different reasons for behaving in different ways. RGT is based on the idea that ‘many attitudes and beliefs get installed in the minds of social actors by their taking some persons or groups as a natural reference, given the situation and questions the actors are exposed to’ (Boudon, 1991: 520). This typology of aspirations to group membership, based on a binary categorisation of group eligibility (‘eligible’ vs ‘non-eligible’) helps to explain how and why ‘respondent standard deviance and variance’ occurs in programmes and often can materialise in focus group discussions.

**Table 1.** Merton’s reference group theory and membership aspirations according to Pawson (2010).

Attitude towards membership	Eligible for membership	Ineligible for membership
Aspire to belong	1. Candidate for membership	2. Marginal person
Indifferent to affiliation	3. Potential member	4. Detached non-member
Motivated not to belong	5. Autonomous non-member	6. Antagonistic non-member

## Reference group theory and sub-group analysis

To illustrate the benefits of group conversations for theory-development by supporting sub-group examination, I will refer to a focus group discussion conducted for the RE previously mentioned (Mirzoev et al. 2015) examining a maternal health programme in Nigeria offering conditional cash transfers to increase healthcare utilisation. CCT programmes became popular in the 1990s and are now present in most low and middle income countries. They transfer cash to people in poverty conditioned to a pre-stipulated behaviour often related to child outcomes (e.g. healthcare utilisation, vaccinations, schooling). Many social science theories accumulate in reward programmes (Fiszbein and Schady, 2009; Wolf et al., 2013) such as self-efficacy theory, self-determination theory (intrinsic and extrinsic motivation); and theories looking at the influence of the setting and context such as the family, the village and so on (e.g. bioecological systems theory). A focus group with eight women who attended the health centre during the CCT programme in Nigeria was conducted during the early stages of the RE. The initial group introductions illustrated how even when the group seemed relatively homogeneous (eight female petty traders and/or seamstress who had given birth in the same healthcare facility), when asked ‘What motivates you to seek healthcare in this health facility?’, five of them offered diverse opinions (e.g. spiritual similarity, affordability, delayed payment facilities, staff attitude towards women, clinical expertise). After listening to their initial replies, the variables of interest to the realist evaluator were no longer ‘women’s occupation’. Instead, theory-relevant variables had been gleaned: ‘women who cannot afford to pay for healthcare fees’, ‘women whose religion is similar to healthcare staff’, ‘women who need delayed payment’ became significant.

The realist evaluator stimulated theorisation by following up with a targeted question about contextual barriers to motivation (‘Are there any difficulties/challenges you experience in this facility?’), knowing that exogenous motivation theories explain how contextual influences (e.g. extrinsic factors such as resource availability and wider social contexts) influence motivation and endogenous theories examine psychological mechanisms within individuals (e.g. intrinsic factors such as self-determination). This prompted a deeper group deliberation, which resulted in a key contextual factor being elucidated:

Participant 1: Some say that whenever they come here, the doctor is not always around. That’s why some persons don’t come to this facility.

Participant 2: There are persons that when they have some certain kind of problems, they will be afraid. Even during ante-natal, they register where there is a doctor so that it can be easy for them. That is why we need a permanent doctor in this facility.

As explained before, in Nigeria, the presence of a permanent physician in rural public maternal healthcare facilities seemed a key driver for some women (i.e. those with previous health conditions, those who can afford private healthcare), who according to Merton’s RGT were not ‘motivated to belong’ to the group of women lured to this facility by the financial rewards, dismissed it and ended up going somewhere else. The importance of a permanent physician potentially generates a new group of significant theory-driven variables ‘women with previous health needs requiring medical monitoring’, ‘women who can attend facilities with a doctor even if they are more expensive or further away’, ‘women who are healthy but scared of delivery’, ‘women with previous bad experiences of giving birth in facilities without physicians’, etc.

While CCT seemed to be targeted at individual level processes (e.g. self-efficacy, motivation, autonomy), institutional contexts (e.g. schools, healthcare facilities) are also an important community-level moderator of programme impacts (Wolf et al., 2013: 10). A key factor impacting healthcare services utilisation and health outcomes is the available supply and the quality of services. CCT are often addressed at the demand side (financial incentives to lure patients in), but if the supply side is not approached, the effectiveness of the programme will be affected. Consequently, the evaluator’s hypothesis (pregnant women’s motivation to utilise healthcare is influenced by perceptions of clinical safety) is moderated by institutional contextual factors (e.g. centres with full-time physicians, with affordable prices and/or delayed payment facilities). This hypothesis builds momentum for the realist evaluator when another participant expanded on how newborn safety (and not cash) is a key driver when choosing a place to give birth. The process of contrasting, comparing and sharing notes within the group starts; and group intelligence develops, through deliberation, consensus and dissensus, and exploring meanings for others alongside reporting their own individual experiences. These claims were refined, triangulated and consolidated with other data collection methods.

Nevertheless, these components of working hypotheses, although they are systematically confirmed with diverse empirical data, are not middle-range theory yet. RGT was not used to guide the focus group deliberation process, instead, RGT positions (see Table 1) are used here as a supplementary post-fieldwork analytical tool to explain why women from different sub-groups (i.e. high-risk pregnancies, first-time mothers, those who lack social support in their communities, those with lower socio-economic status) behaved differently to the promise of the financial incentive. For instance, those who were already attending maternal and child healthcare services in that facility before the programme offered incentives were ‘indifferent to affiliation’ but still enjoyed and accessed the cash transfers. Others attending maternity care in different healthcare facilities may have moved temporarily to this one because of the CCT. These phenomena are well-known unintended consequences of rewards programmes such as displacement and short-lived outcomes (Hood, 2006).

RGT supports the causality explanations and a middle-range sociological theory starts to be formed. For example, a direct question in the focus group discussion about subgroups leads the evaluator down the ‘rabbit hole’ of one subgroup: those who are not familiar with the healthcare institution where the CCT in exchange of maternal care utilisation is offered. Practical instances of volunteer health workers (VHW) support (a programme activity implemented alongside CCTs), where trust was built through practical help and direction setting (i.e. transporting women to health centres, giving them free Mama kits^2^), were mentioned during the focus group discussion. The diversity of women’s motivations is embedded in macro–meso–micro-structural circumstances, which can be mobilised or not by other programme activities and resources. During the focus group deliberations, these ideas were gleaned and they were developed later on after further analysis and data collection. The continuous iterative feedback loop (Robert et al., 2017) between the theoretical and empirical literature to capture the intricate relationships between trust, staff and user motivations in this evaluation has been reported elsewhere (Ezumah et al., 2022; Mirzoev et al., 2020).

Table 2 summarises how other programme resources (i.e. human resources such as VHW) could encourage women ‘ineligible for membership’ to belong to the group as ‘antagonistic non-members’ by raising awareness and befriending them. Through practical support and direction setting, women became detached non-members. Equally important, they also coached them through fears about clinical expertise in the facility and treatment safety (i.e. child immunisation). They would build trust in the communities and those women became ‘candidates for membership’ and were more likely to access the healthcare facility for maternal services.

**Table 2.** Reference group theory, conditional cash transfers and the role of VHW.

Attitude towards membership	Eligible for membership	Ineligible for membership
Aspire to belong (Women who don’t attend the hospital but would prefer to do so)	Candidate for membership (Trust in clinical expertise)	Marginal person (VHW and others coach through fear) 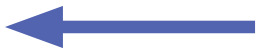 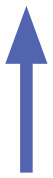
Indifferent to affiliation: (Women already attending the hospital)	Potential member	Detached non-member (VHW practical support and direction setting) 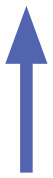
Motivated not to belong (Women who don’t attend the hospital and prefer to deliver and control the pregnancy by traditional means)	Autonomous non-member	Antagonistic non-member (VHW awareness and befriending) 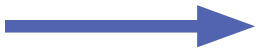

VHW: Volunteer health workers.

In summary, the unique focus group deliberation allows for examination of how programmes impact sub-groups disparately, and/or how mechanisms may or may not be less likely to be triggered in certain circumstances, for certain sub-groups, with certain sub-characteristics. Realist focus groups are conducted within a programme evaluation context, where the individual group member stories are not the aim of the encounter. Instead, subgroup reasoning is extracted through group intelligence and deliberation to illuminate the complex causality embedded in underlying mechanisms, contexts and programme intended and unintended outcomes.

## Conclusion

Recent calls in the social sciences promote more transparent and rigorous focus group practices (Cyr, 2016). Although in 1946, Merton and Kendall formalised ‘focused groups’ as tools for theory development, decades of adaption and readaptation in many disciplines within and outside evaluation diluted the original purpose of testing and refining hypotheses. In evaluation science, however, the BASR advice on ‘focussed’ group discussion, with the help of theory and compared and contrasted with other mixed-methods, is followed. In this way, qualitative methods in evaluation research continue to support the construction of middle-range theory. As Fetterman (2003: 47) noted, the ‘conundrum of linking theory and practice is common to many fields’ and not only evaluation. Having emerged in the disciplines of political opinion polling and consumer research, the unproblematic status of data collected through focus group discussions in realist investigations is surprising, given that knowledge claims in realist studies are routinely subjected to intense analytical academic group scrutiny.

This article demonstrates how focus group deliberations in REs can help disentangle how programmes work differently in infinite contexts. As Merton (1987) noted, focus groups cannot stand alone but they should be key tools in mixed-methods evaluations. As demonstrated with the example of the RE approach, theory-driven data collection can help transform hypotheses into middle-range theory. In the same way that Merton et al. did in the 1940s, ‘focused’ group conversations are still successfully used not only for generating theory but they can also assist in testing it and refining it. Profound methodological ideas of the 1940s survive obliteration and live on.

## Supplemental Material

sj-docx-1-evi-10.1177_13563890221124637 – Supplemental material for Conducting focus groups in realist evaluation Click here for additional data file. Supplemental material, sj-docx-1-evi-10.1177_13563890221124637 for Conducting focus groups in realist evaluation by Ana Manzano in Evaluation

## Notes

1. Henceforth, in this article, the role of Bureau of Applied Social Research’s (BASR) female researchers will be acknowledged by referring to ‘Merton et al.’ or “Merton and others” to refer to the work on focus groups developed by the BASR team of researchers.

2. Mama kits contain essential materials for delivery and newborn care that must be brought into healthcare facilities by women themselves to be used during delivery. They were freely given to all pregnant women who attended health facilities participating in the conditional cash transfers (CCT) programme.
