# Supplementary material for: Conducting focus groups in realist evaluation
Source: Evaluation (Lond). 2022 Sep 15;28(4):406–25. doi: 10.1177/13563890221124637 (PMC9530522; doi:10.1177/13563890221124637)
Supplement: sj-docx-1-evi-10.1177_13563890221124637 – Supplemental material for Conducting focus groups in realist evaluation [file sj-docx-1-evi-10.1177_13563890221124637.docx]

**Supplementary Table**

SEARCHES

| 1 (realist adj (evaluat* and focus group*)).ti, ab. 111 |
| --- |
| 2 (realist adj (evaluat* and stakeholder*)).ti, ab. 135 |
| 3 (realist adj (synthes* and focus group*)).ti, ab. 35 |
| 4 (realist adj (synthes* and stakeholder*)).ti, ab. 96 |
| 5 (realist adj (review* and focus group*)).ti, ab. 74 |
| 6 (realist adj (review* and stakeholder*)).ti, ab. 152 |
| 7 1 or 2 or 3 or 4 or 5 or 6 |

Searched in: Scopus
